# 4-(4-Bromo­phenyl)-2-methyl-2,6-di­phenyl-2*H*-thio­pyran

**DOI:** 10.1107/S1600536809005959

**Published:** 2009-02-25

**Authors:** Hossein Rahmani, Hooshang Pirelahi, Seik Weng Ng

**Affiliations:** a Institute of Chemical Industries, Iranian Research Organization for Science and Technology, PO Box 15815-358, Tehran, Iran; b Department of Chemistry, College of Science, University of Tehran, PO Box 13145-143, Tehran, Iran; c Department of Chemistry, University of Malaya, 50603 Kuala Lumpur, Malaysia

## Abstract

The six-membered thio­pyran ring in the title compound, C_24_H_19_BrS, adopts an approximate envelope conformation, with the S atom displaced by 0.26 (1) Å and the 2-methyl­ene C atom by −0.54 (1) Å from the plane of the other four *sp* ^2^-hydridized C atoms. The methyl substituent on the methyl­ene carbon lies in a pseudo-axial position with the phenyl ring in a pseudo-equatorial position.

## Related literature

For the background to 4-alkyl-2,4,6-triaryl-4*H*-thio­pyrans, see: Rahmani *et al.* (2009 ▶). For the general synthesis from a Grignard reaction, see: Suld & Price (1962 ▶).
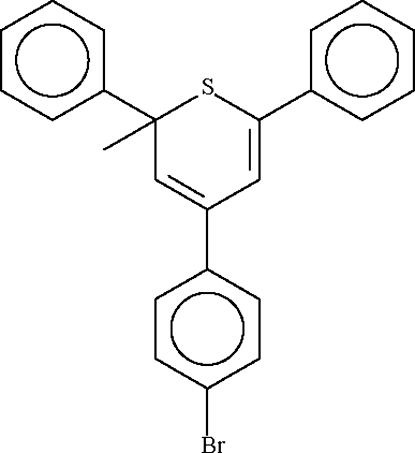


## Experimental

### 

#### Crystal data

C_24_H_19_BrS
*M* *_r_* = 419.36
Orthorhombic, 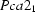

*a* = 23.3348 (8) Å
*b* = 5.9991 (2) Å
*c* = 13.6866 (5) Å
*V* = 1916.0 (1) Å^3^
*Z* = 4
Mo *K*α radiation
μ = 2.26 mm^−1^
*T* = 115 K
0.40 × 0.15 × 0.05 mm

#### Data collection

Bruker SMART APEX diffractometer
Absorption correction: multi-scan (*SADABS*; Sheldrick, 1996 ▶) *T*_min_ = 0.465, *T*_max_ = 0.895
16215 measured reflections
4407 independent reflections
3017 reflections with *I* > 2σ(*I*)
*R*_int_ = 0.093

#### Refinement

*R*[*F* ^2^ > 2σ(*F* ^2^)] = 0.075
*wR*(*F* ^2^) = 0.190
*S* = 1.09
4407 reflections
236 parameters
145 restraints
H-atom parameters constrained
Δρ_max_ = 1.53 e Å^−3^
Δρ_min_ = −0.91 e Å^−3^
Absolute structure: Flack (1983 ▶), 2108 Friedel pairs
Flack parameter: 0.01 (2)

### 

Data collection: *APEX2* (Bruker, 2008 ▶); cell refinement: *SAINT* (Bruker, 2008 ▶); data reduction: *SAINT*; program(s) used to solve structure: *SHELXS97* (Sheldrick, 2008 ▶); program(s) used to refine structure: *SHELXL97* (Sheldrick, 2008 ▶); molecular graphics: *X-SEED* (Barbour, 2001 ▶); software used to prepare material for publication: *publCIF* (Westrip, 2009 ▶).

## Supplementary Material

Crystal structure: contains datablocks global, I. DOI: 10.1107/S1600536809005959/sj2581sup1.cif

Structure factors: contains datablocks I. DOI: 10.1107/S1600536809005959/sj2581Isup2.hkl

Additional supplementary materials:  crystallographic information; 3D view; checkCIF report

## Figures and Tables

**Fig. 1.**
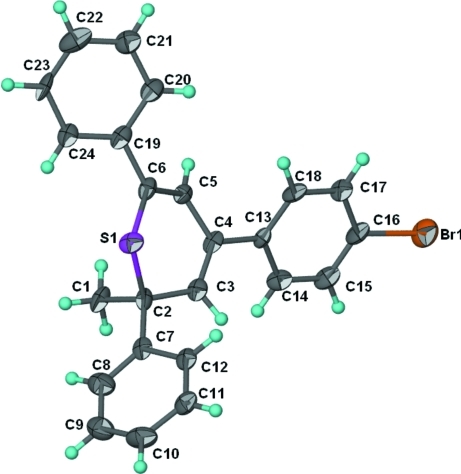
Thermal ellisoid plot (Barbour, 2001) of C24H19BrS; probability levels are set at 70% and H-atoms are drawn as spheres of arbitrary radius.

## supplementary crystallographic information

### Experimental

The compound was obtained as the rearranged product from the reaction of methyl magnesium bromide and 4-(4-bromophenyl)-2,6-diphenyl thiopyrylium perchlorate in dry ether under an argon atmosphere according to a reported method (Suld & Price, 1962). The product was isolated by TLC on neutral alumina (petroleum ether 40–60 °C) and purified by recrystalization from ethanol.

### Refinement

Carbon-bound H-atoms were placed in calculated positions (C–H 0.95 to 0.98 Å) and were included in the refinement in the riding model approximation, with *U*(H) set to 1.2 to 1.5*U*(C).

The final difference Fourier map had a large peak/deep hole in the vicinity of the bromine.

### Crystal data

**Table:**

C_24_H_19_BrS	*F*(000) = 856
*M**_r_* = 419.36	*D*_x_ = 1.454 Mg m^−^^3^
Orthorhombic, *P**c**a*2_1_	Mo *K*α radiation, λ = 0.71073 Å
Hall symbol: P 2c -2ac	Cell parameters from 2506 reflections
*a* = 23.3348 (8) Å	θ = 2.3–22.9°
*b* = 5.9991 (2) Å	µ = 2.26 mm^−^^1^
*c* = 13.6866 (5) Å	*T* = 115 K
*V* = 1916.0 (1) Å^3^	Prism, pale yellow
*Z* = 4	0.40 × 0.15 × 0.05 mm

### Data collection

**Table:**

Bruker SMART APEX diffractometer	4407 independent reflections
Radiation source: fine-focus sealed tube	3017 reflections with *I* > 2σ(*I*)
graphite	*R*_int_ = 0.093
ω scans	θ_max_ = 27.5°, θ_min_ = 1.8°
Absorption correction: multi-scan (*SADABS*; Sheldrick, 1996)	*h* = −30→29
*T*_min_ = 0.465, *T*_max_ = 0.895	*k* = −7→7
16215 measured reflections	*l* = −17→17

### Refinement

**Table:**

Refinement on *F*^2^	Secondary atom site location: difference Fourier map
Least-squares matrix: full	Hydrogen site location: inferred from neighbouring sites
*R*[*F*^2^ > 2σ(*F*^2^)] = 0.075	H-atom parameters constrained
*wR*(*F*^2^) = 0.190	*w* = 1/[σ^2^(*F*_o_^2^) + (0.0672*P*)^2^ + 10.0175*P*] where *P* = (*F*_o_^2^ + 2*F*_c_^2^)/3
*S* = 1.09	(Δ/σ)_max_ = 0.001
4407 reflections	Δρ_max_ = 1.53 e Å^−^^3^
236 parameters	Δρ_min_ = −0.91 e Å^−^^3^
145 restraints	Absolute structure: Flack (1983), 2108 Friedel pairs
Primary atom site location: structure-invariant direct methods	Flack parameter: 0.01 (2)

### Fractional atomic coordinates and isotropic or equivalent isotropic displacement parameters (Å^2^)

**Table:**

	*x*	*y*	*z*	*U*_iso_*/*U*_eq_	
Br1	0.55183 (4)	0.84726 (15)	0.50001 (7)	0.0381 (3)	
S1	0.76405 (9)	−0.1684 (4)	0.84041 (14)	0.0245 (4)	
C1	0.7867 (4)	−0.2735 (13)	0.6484 (6)	0.0260 (18)	
H1A	0.8005	−0.2273	0.5838	0.039*	
H1B	0.7469	−0.3246	0.6431	0.039*	
H1C	0.8107	−0.3952	0.6730	0.039*	
C2	0.7898 (3)	−0.0738 (13)	0.7196 (5)	0.0198 (16)	
C3	0.7503 (3)	0.1075 (12)	0.6851 (5)	0.0206 (17)	
H3	0.7669	0.2372	0.6571	0.025*	
C4	0.6924 (3)	0.0971 (13)	0.6915 (5)	0.0191 (16)	
C5	0.6643 (3)	−0.0773 (13)	0.7470 (5)	0.0183 (16)	
H5	0.6252	−0.1083	0.7332	0.022*	
C6	0.6907 (4)	−0.1988 (12)	0.8174 (5)	0.0204 (17)	
C7	0.8509 (3)	0.0024 (14)	0.7371 (5)	0.0202 (17)	
C8	0.8986 (4)	−0.1115 (16)	0.7095 (6)	0.032 (2)	
H8	0.8937	−0.2494	0.6764	0.039*	
C9	0.9534 (4)	−0.0388 (16)	0.7267 (6)	0.033 (2)	
H9	0.9852	−0.1234	0.7039	0.040*	
C10	0.9623 (4)	0.1547 (18)	0.7762 (7)	0.034 (2)	
H10	1.0000	0.2016	0.7922	0.041*	
C11	0.9148 (4)	0.2837 (15)	0.8034 (6)	0.0263 (19)	
H11	0.9206	0.4238	0.8342	0.032*	
C12	0.8604 (4)	0.2107 (13)	0.7863 (5)	0.0225 (17)	
H12	0.8287	0.2979	0.8069	0.027*	
C13	0.6567 (3)	0.2668 (12)	0.6448 (5)	0.0168 (15)	
C14	0.6740 (4)	0.3678 (14)	0.5562 (5)	0.0236 (17)	
H14	0.7081	0.3187	0.5253	0.028*	
C15	0.6422 (3)	0.5366 (13)	0.5137 (6)	0.0274 (18)	
H15	0.6552	0.6058	0.4553	0.033*	
C16	0.5923 (4)	0.6028 (14)	0.5560 (6)	0.0276 (19)	
C17	0.5729 (4)	0.5021 (14)	0.6414 (5)	0.0223 (17)	
H17	0.5376	0.5474	0.6698	0.027*	
C18	0.6048 (4)	0.3370 (14)	0.6844 (6)	0.0230 (17)	
H18	0.5912	0.2691	0.7427	0.028*	
C19	0.6600 (4)	−0.3593 (13)	0.8826 (5)	0.0222 (17)	
C20	0.6058 (4)	−0.2976 (14)	0.9198 (6)	0.0258 (19)	
H20	0.5896	−0.1564	0.9046	0.031*	
C21	0.5766 (4)	−0.4471 (14)	0.9787 (5)	0.0284 (19)	
H21	0.5393	−0.4106	1.0017	0.034*	
C22	0.6007 (4)	−0.6463 (15)	1.0043 (8)	0.0363 (19)	
H22	0.5808	−0.7436	1.0473	0.044*	
C23	0.6542 (4)	−0.7082 (13)	0.9679 (6)	0.0259 (19)	
H23	0.6702	−0.8489	0.9843	0.031*	
C24	0.6841 (4)	−0.5623 (13)	0.9073 (5)	0.0214 (17)	
H24	0.7209	−0.6021	0.8831	0.026*	

### Atomic displacement parameters (Å^2^)

**Table:**

	*U*^11^	*U*^22^	*U*^33^	*U*^12^	*U*^13^	*U*^23^
Br1	0.0382 (5)	0.0361 (4)	0.0401 (5)	0.0017 (4)	−0.0071 (5)	0.0148 (5)
S1	0.0254 (10)	0.0295 (11)	0.0186 (9)	−0.0016 (9)	−0.0016 (8)	0.0090 (9)
C1	0.041 (5)	0.014 (4)	0.023 (4)	−0.005 (3)	−0.002 (4)	0.000 (3)
C2	0.029 (4)	0.015 (3)	0.016 (3)	0.004 (3)	0.001 (3)	0.001 (3)
C3	0.027 (4)	0.021 (4)	0.014 (3)	0.001 (3)	−0.003 (3)	0.005 (3)
C4	0.031 (4)	0.018 (4)	0.008 (3)	−0.002 (3)	−0.003 (3)	0.006 (3)
C5	0.023 (4)	0.016 (4)	0.016 (3)	−0.002 (3)	0.001 (3)	0.000 (3)
C6	0.031 (4)	0.015 (4)	0.015 (3)	0.000 (3)	−0.001 (3)	0.002 (3)
C7	0.031 (4)	0.020 (4)	0.010 (3)	0.004 (3)	0.000 (3)	0.007 (3)
C8	0.028 (4)	0.041 (5)	0.028 (4)	0.004 (4)	0.003 (3)	0.010 (4)
C9	0.028 (4)	0.042 (5)	0.028 (4)	0.005 (4)	0.005 (4)	0.005 (4)
C10	0.020 (4)	0.047 (5)	0.036 (4)	−0.007 (4)	−0.004 (3)	0.010 (4)
C11	0.032 (5)	0.023 (4)	0.024 (4)	−0.001 (4)	−0.002 (4)	0.010 (3)
C12	0.033 (4)	0.020 (4)	0.015 (3)	0.002 (3)	0.005 (3)	0.004 (3)
C13	0.022 (4)	0.014 (3)	0.015 (3)	−0.002 (3)	−0.004 (3)	0.004 (3)
C14	0.025 (4)	0.030 (4)	0.016 (3)	−0.004 (3)	−0.002 (3)	0.004 (3)
C15	0.036 (4)	0.029 (4)	0.017 (4)	−0.006 (3)	−0.001 (3)	0.010 (3)
C16	0.035 (5)	0.024 (4)	0.024 (4)	−0.004 (3)	0.002 (4)	0.012 (3)
C17	0.029 (4)	0.025 (4)	0.013 (3)	0.001 (3)	−0.001 (3)	0.007 (3)
C18	0.025 (4)	0.026 (4)	0.017 (3)	−0.007 (3)	−0.001 (3)	0.010 (3)
C19	0.032 (4)	0.019 (4)	0.016 (3)	−0.004 (3)	−0.004 (3)	0.002 (3)
C20	0.032 (4)	0.025 (4)	0.020 (3)	−0.002 (3)	−0.001 (3)	0.007 (3)
C21	0.032 (4)	0.032 (4)	0.022 (4)	−0.002 (3)	−0.001 (3)	0.006 (3)
C22	0.043 (4)	0.041 (4)	0.025 (3)	−0.018 (4)	−0.006 (5)	0.009 (4)
C23	0.046 (5)	0.010 (4)	0.022 (4)	−0.003 (3)	−0.006 (3)	0.006 (3)
C24	0.031 (4)	0.020 (4)	0.013 (3)	0.000 (3)	−0.002 (3)	0.002 (3)

### Geometric parameters (Å, °)

**Table:**

Br1—C16	1.905 (8)	C11—C12	1.363 (12)
S1—C6	1.750 (8)	C11—H11	0.9500
S1—C2	1.849 (8)	C12—H12	0.9500
C1—C2	1.547 (11)	C13—C18	1.391 (11)
C1—H1A	0.9800	C13—C14	1.414 (10)
C1—H1B	0.9800	C14—C15	1.383 (11)
C1—H1C	0.9800	C14—H14	0.9500
C2—C3	1.501 (10)	C15—C16	1.360 (12)
C2—C7	1.516 (11)	C15—H15	0.9500
C3—C4	1.356 (11)	C16—C17	1.392 (11)
C3—H3	0.9500	C17—C18	1.373 (11)
C4—C5	1.448 (10)	C17—H17	0.9500
C4—C13	1.463 (10)	C18—H18	0.9500
C5—C6	1.357 (11)	C19—C24	1.383 (11)
C5—H5	0.9500	C19—C20	1.414 (12)
C6—C19	1.495 (11)	C20—C21	1.385 (11)
C7—C8	1.361 (12)	C20—H20	0.9500
C7—C12	1.436 (11)	C21—C22	1.366 (13)
C8—C9	1.372 (12)	C21—H21	0.9500
C8—H8	0.9500	C22—C23	1.395 (13)
C9—C10	1.360 (14)	C22—H22	0.9500
C9—H9	0.9500	C23—C24	1.393 (11)
C10—C11	1.402 (13)	C23—H23	0.9500
C10—H10	0.9500	C24—H24	0.9500
			
C6—S1—C2	100.9 (4)	C11—C12—C7	120.3 (8)
C2—C1—H1A	109.5	C11—C12—H12	119.8
C2—C1—H1B	109.5	C7—C12—H12	119.8
H1A—C1—H1B	109.5	C18—C13—C14	116.9 (7)
C2—C1—H1C	109.5	C18—C13—C4	122.4 (6)
H1A—C1—H1C	109.5	C14—C13—C4	120.7 (7)
H1B—C1—H1C	109.5	C15—C14—C13	121.5 (8)
C3—C2—C7	114.1 (7)	C15—C14—H14	119.3
C3—C2—C1	109.5 (6)	C13—C14—H14	119.3
C7—C2—C1	112.2 (7)	C16—C15—C14	119.5 (7)
C3—C2—S1	107.7 (5)	C16—C15—H15	120.2
C7—C2—S1	104.9 (5)	C14—C15—H15	120.2
C1—C2—S1	108.1 (5)	C15—C16—C17	120.7 (8)
C4—C3—C2	123.9 (7)	C15—C16—Br1	118.6 (6)
C4—C3—H3	118.0	C17—C16—Br1	120.7 (7)
C2—C3—H3	118.0	C18—C17—C16	119.8 (8)
C3—C4—C5	121.2 (7)	C18—C17—H17	120.1
C3—C4—C13	120.5 (7)	C16—C17—H17	120.1
C5—C4—C13	118.3 (7)	C17—C18—C13	121.6 (7)
C6—C5—C4	123.7 (7)	C17—C18—H18	119.2
C6—C5—H5	118.1	C13—C18—H18	119.2
C4—C5—H5	118.1	C24—C19—C20	120.4 (7)
C5—C6—C19	123.6 (8)	C24—C19—C6	121.2 (8)
C5—C6—S1	121.0 (6)	C20—C19—C6	118.4 (7)
C19—C6—S1	115.3 (6)	C21—C20—C19	118.7 (8)
C8—C7—C12	116.1 (8)	C21—C20—H20	120.6
C8—C7—C2	125.1 (8)	C19—C20—H20	120.6
C12—C7—C2	118.8 (7)	C22—C21—C20	120.9 (8)
C7—C8—C9	123.8 (9)	C22—C21—H21	119.6
C7—C8—H8	118.1	C20—C21—H21	119.6
C9—C8—H8	118.1	C21—C22—C23	120.6 (9)
C10—C9—C8	119.9 (9)	C21—C22—H22	119.7
C10—C9—H9	120.1	C23—C22—H22	119.7
C8—C9—H9	120.1	C24—C23—C22	119.6 (8)
C9—C10—C11	118.9 (8)	C24—C23—H23	120.2
C9—C10—H10	120.5	C22—C23—H23	120.2
C11—C10—H10	120.5	C19—C24—C23	119.7 (8)
C12—C11—C10	120.8 (9)	C19—C24—H24	120.2
C12—C11—H11	119.6	C23—C24—H24	120.2
C10—C11—H11	119.6		
			
C6—S1—C2—C3	46.9 (6)	C2—C7—C12—C11	179.8 (7)
C6—S1—C2—C7	168.8 (5)	C3—C4—C13—C18	−146.5 (8)
C6—S1—C2—C1	−71.3 (6)	C5—C4—C13—C18	31.7 (11)
C7—C2—C3—C4	−160.3 (7)	C3—C4—C13—C14	33.4 (11)
C1—C2—C3—C4	73.0 (9)	C5—C4—C13—C14	−148.5 (7)
S1—C2—C3—C4	−44.3 (9)	C18—C13—C14—C15	3.2 (12)
C2—C3—C4—C5	10.0 (11)	C4—C13—C14—C15	−176.6 (7)
C2—C3—C4—C13	−171.9 (7)	C13—C14—C15—C16	−2.1 (13)
C3—C4—C5—C6	19.9 (12)	C14—C15—C16—C17	−0.2 (13)
C13—C4—C5—C6	−158.2 (7)	C14—C15—C16—Br1	176.4 (6)
C4—C5—C6—C19	172.4 (7)	C15—C16—C17—C18	1.2 (13)
C4—C5—C6—S1	−5.3 (11)	Br1—C16—C17—C18	−175.3 (7)
C2—S1—C6—C5	−26.8 (7)	C16—C17—C18—C13	0.0 (13)
C2—S1—C6—C19	155.3 (6)	C14—C13—C18—C17	−2.2 (12)
C3—C2—C7—C8	−136.8 (8)	C4—C13—C18—C17	177.7 (8)
C1—C2—C7—C8	−11.5 (11)	C5—C6—C19—C24	139.8 (8)
S1—C2—C7—C8	105.6 (8)	S1—C6—C19—C24	−42.3 (9)
C3—C2—C7—C12	43.0 (9)	C5—C6—C19—C20	−40.9 (11)
C1—C2—C7—C12	168.3 (6)	S1—C6—C19—C20	136.9 (7)
S1—C2—C7—C12	−74.6 (7)	C24—C19—C20—C21	−1.9 (12)
C12—C7—C8—C9	0.4 (12)	C6—C19—C20—C21	178.8 (7)
C2—C7—C8—C9	−179.8 (8)	C19—C20—C21—C22	2.9 (13)
C7—C8—C9—C10	1.9 (13)	C20—C21—C22—C23	−3.0 (14)
C8—C9—C10—C11	−4.1 (13)	C21—C22—C23—C24	2.0 (13)
C9—C10—C11—C12	4.2 (13)	C20—C19—C24—C23	1.0 (11)
C10—C11—C12—C7	−1.9 (12)	C6—C19—C24—C23	−179.7 (7)
C8—C7—C12—C11	−0.4 (11)	C22—C23—C24—C19	−1.0 (12)
